# ChrA_SO_, the chromate efflux pump of *Shewanella oneidensis*, improves chromate survival and reduction

**DOI:** 10.1371/journal.pone.0188516

**Published:** 2017-11-22

**Authors:** Hiba Baaziz, Cyril Gambari, Anne Boyeldieu, Amine Ali Chaouche, Radia Alatou, Vincent Méjean, Cécile Jourlin-Castelli, Michel Fons

**Affiliations:** 1 Unité de Bioénergétique et Ingénierie des Protéines, Institut de Microbiologie de la Méditerranée, Aix-Marseille Université, Centre National de la Recherche Scientifique, Marseille, France; 2 Laboratoire de Biologie Moléculaire et Cellulaire, Université des Frères Mentouri Constantine 1, Constantine, Algeria; Centre National de la Recherche Scientifique, Aix-Marseille Université, FRANCE

## Abstract

The chromate efflux pump encoding gene *chrA*_*SO*_ was identified on the chromosome of *Shewanella oneidensis* MR1. Although *chrA*_*SO*_ is expressed without chromate, its expression level increases when Cr(VI) is added. When deleted, the resulting mutant Δ*chrA*_*SO*_ exhibits a chromate sensitive phenotype compared to that of the wild-type strain. Interestingly, heterologous expression of *chrA*_*SO*_ in *E*. *coli* confers resistance to high chromate concentration. Moreover, expression of *chrA*_*SO*_ in *S*. *oneidensis* and *E*. *coli* significantly improves Cr(VI) reduction. This effect could result either from extracytoplasmic chromate reduction or from a better cell survival leading to enhanced Cr(VI) reduction.

## Introduction

The transition metal chromium has different oxidation states, but only two of them are stable, namely the trivalent Cr(III) and the hexavalent Cr(VI) forms. The Cr(III) form which is relatively insoluble is considered to be poorly toxic since it can hardly penetrate the cells. The Cr(VI) form (chromate and dichromate) is soluble and can be conveyed through cell membranes by different transporters, making it a very toxic compound for both eukaryotic and prokaryotic cells [1].

Chromate, which is chemically and structurally similar to sulfate, is thought to enter the bacterial cells mainly via sulfate ABC transporters. Once inside the cells, Cr(VI) can be reduced, either enzymatically or not, giving rise to intermediates which are responsible for various damages to DNA and cellular components. Some of these alterations are due to the generation of reactive oxygen species (ROS). When present inside the cells, Cr(III), the final product of Cr(VI) reduction, causes toxic effects on both DNA and proteins by binding respectively to phosphate, and to carboxyl and thiol groups [2].

Bacteria have developed different strategies to resist chromate [2,3]. One strategy is to limit its cell entry by reducing Cr(VI) to Cr(III) extracellularly. Reduction can be achieved anaerobically by membrane bound reductases like flavin reductases, cytochromes and hydrogenases that are part of electron transport systems using chromate as electron acceptor. In sulfate-reducing bacteria for example, extracellular Cr(VI) reduction can be carried out both enzymatically and chemically by oxidation of the H_2_S generated during anaerobic respiration [4,5].

A second strategy is to reduce aerobically the intracellular Cr(VI) [6]. The best known cytosolic proteins involved in this reduction are ChrR of *Pseudomonas putida* and YieF of *Escherichia coli* [7,8]. Both enzymes share sequence homologies and belong to the class I chromate reductases. Nevertheless their mechanisms of action are different. YieF directly reduces Cr(VI) to Cr(III) through a four-electron transfer reaction while ChrR proceeds by combining one- and two-electron transfer steps [7]. As a result, ChrR generates more ROS than YieF during chromate reduction. The class II chromate reductases could require NAD(P)H as an electron donor and generally possess a nitroreductase activity. One of them, NfsA from *E*. *coli*, was characterized and shown to increase chromate tolerance when overproduced [9]. Intracellular chromate reduction is often associated with mechanisms to detoxify the cell from the ROS and to repair the lesions, caused in particular to DNA [10,11].

A third strategy to deal with chromate toxicity is to rapidly extrude it from the cell cytoplasm by the mean of chromate efflux pump. The ChrA proteins of *Pseudomonas aeruginosa* and *Cupriavidus metallidurans* were the first proteins proved to be involved in this process [12,13]. Thereafter, several ChrA homologues were identified based on genome sequence analysis and grouped into a large family, called the Chromate ion transporter (CHR) family [14]. This latter was further divided into two subfamilies on the basis of the protein length. One subfamily contains proteins with a sequence length of about 180 amino acids, named short-chain CHR (SCHR), and bearing only one chromate transporter domain. The proteins of the second subfamily, containing two chromate transporter domains, are larger (around 400 amino acids) and called long-chain CHR (LCHR). The ChrA proteins possess several transmembrane regions and are encoded by genes that can be found either on chromosome or on plasmid. Some bacteria possess several *chrA* genes associated or not with other genes involved in chromate resistance or regulation (like *chrB*, *chrC* or *chrF*, found for example in *C*. *metallidurans*, *Ochrobactrum tritici* and *Shewanella sp*. strain ANA-3) [14]. These examples are not exhaustive and different strategies can co-exist in a same bacterium.

*Shewanella oneidensis* is an aquatic bacterium that belongs to the ɣ-Proteobacteria class. It can utilize a wide range of terminal electron acceptors including fumarate, nitrate, TMAO, as well as oxidized metals including Fe(III) and Mn(IV). This respiratory versatility makes it a key player for bioremediation [15]. The capability of *S*. *oneidensis* MR-1 to resist to and/or reduce Cr(VI) has been explored in various conditions, pointing out the involvement of different mechanisms for these functions [16–18]. A transcriptomic analysis revealed that more than 80 genes were upregulated when strain MR-1 was under Cr(VI)-reduction condition [19]. Moreover, the response regulator SO2426 was shown to control the expression of genes involved in the relationship between Fe homeostasis and Cr(VI)-induced stress tolerance [20]. Concerning reduction, MtrC and OmcA, two extracellular decaheme cytochromes located in the outer membrane of the cells and previously known to be involved in Fe(III) reduction, were characterized as terminal Cr(VI) reductases *in vitro* as well as *in vivo* [18,21]. A protein homologous to ChrR of *P*. *putida* was also identified in *S*. *oneidensis* (SO3585), but its role in chromate resistance seems not to be crucial since only the initial rate of chromate disappearance is affected in the *so3585*-deleted strain [22].

The aim of this work was to confirm that resistance to chromate in *S*. *oneidensis* MR-1 involves a chromate efflux pump and to evaluate the impact of this efflux pump on the capability of this bacterium to reduce Cr(VI) to the less toxic form Cr(III).

## Materials and methods

### Strains, medium and growth conditions

The *E*. *coli* strains CC118 λpir and 1047/pRK2013 used for conjugation, and MC1061 derivatives used for chromate resistance and reduction assays were routinely grown in Lysogeny Broth (LB) medium at 37°C, or 30°C when specified [23,24].

All *S*. *oneidensis* strains used in this study are derivatives of strain MR1-R (referred as wild-type and used instead of MR-1 to allow counter-selection by rifampicin in the conjugation experiments) and were routinely grown at 28°C in LB under aerobic (agitation) or anaerobic (static) conditions. In the latter case, LB medium was supplemented with 20 mM trimethylamine oxide (TMAO) as electron acceptor. When growth was performed in the presence of chromate, disposable tubes were used and incubated either statically or under agitation. The dissolved oxygen (DO) of the latter condition measured by a Clark electrode was 10%. This condition was then referred as semi-aerobic. Chromate challenge was carried out by supplementing LB medium at the required final concentration with a filter-sterilized stock solution of potassium chromate (K_2_CrO_4_, Sigma-Aldrich).

If required, media were solidified by adding 17 g.L^-1^ agar. When needed, chloramphenicol (Cm) was used at 25 μg.mL^–1^. Growth was determined spectrophotometrically by monitoring changes in optical density at 600 nm compared to the same medium without bacterium (OD_600nm_).

### Chromate resistance assays in *S*. *oneidensis*

*S*. *oneidensis* pre-cultures grown overnight on LB plates were suspended in LB medium and used to inoculate fresh LB medium to an initial OD_600nm_ = 0.2. Cells were then grown until an OD_600nm_ = 0.5 and submitted to two different assays.

For the spot assay, 10-fold serial dilutions of cell cultures were spotted on LB plates supplemented or not with 0.5 mM of chromate and incubated at 28°C. Plates were scanned after 4 days of incubation.

For the viability assay, chromate was added to the cell cultures (OD_600nm_ = 0.5) to a final concentration of 0.2 mM. After 5 hours, cells were appropriately diluted in LB and spread onto LB agar, and incubated at 28°C. The total number of viable cells was estimated based on the number of colony-forming unit (CFU). Results were expressed as the percentage of viable counts measured in these conditions compared to that of the same culture grown in the absence of chromate.

### Cr(VI)-reduction assays in *S*. *oneidensis*

*In vivo* Cr(VI) measurements were carried out on both semi-aerobic and anaerobic cultures. When fresh cultures reached an OD_600nm_ = 0.5, chromate was added to a final concentration of 1 mM. After 2h of incubation at 28°C, cell cultures were centrifuged at 8,000 g for 5 min and residual Cr(VI) concentration present in the supernatant was determined using the S-diphenylcarbazide (DPC) method [25] slightly modified. Briefly, 10, 20, and 50 μL supernatant samples were added in a 2 mL tube containing 1 mL of H_3_PO_4_ 0.5% and the volume of water necessary to obtain a 1960 μL final volume. Forty μL of DPC reagent (5 mg.mL^-1^ in 95% acetone and stored in dark at room temperature) were added, gently mixed and kept at room temperature for 5–10 min. Absorbance was measured at 540 nm. Cr(VI) concentration in the samples was calculated from a standard curve. These experiments were repeated three times.

*In vitro* measurements of chromate reductase activity were performed on crude extracts as described previously [26] with slight modifications. Briefly, wild-type and *chrA*_*SO*_ deleted mutant strains were grown overnight in LB medium at 28°C under agitation. The cells were then harvested, washed with sodium phosphate buffer (pH7) and resuspended in the same buffer before disruption by French press. The cell lysates were then centrifuged and the supernatants (crude extracts) recovered. Protein concentrations were measured using Protein Assay Dye Reagent (Bio-Rad), and bovine serum albumin as standard. Reaction mixtures (1 mL) containing 100 μM of K_2_CrO_4_ [Cr(VI)] and 1.5 mg of proteins (crude extracts) were incubated at 30°C for 30 min. The residual Cr(VI) concentration in the reaction mixture was estimated using the DPC method as described above.

### Construction of plasmid p*chrA*_*SO*_

To evaluate the impact of ChrA_SO_ on chromate resistance and reduction in *E*. *coli*, the *chrA*_*SO*_ open reading frame (*so0986*) was PCR-amplified from MR1-R chromosomal DNA, from upstream of the ATG start codon (with an optimized Shine Dalgarno) to the TAA stop codon, and cloned between the *Sac*I and the *Xba*I restriction sites of the pBAD33 vector, under the control of the inducible P_*ara*_ promoter [27]. The resulting plasmid, called p*chrA*_*SO*_, was introduced into strain MC1061 and controlled by DNA-sequencing (S1 Table).

### Chromate resistance and reduction assays in *E*. *coli*

Pre-cultures of MC1061 derivatives containing either the plasmid p*chrA*_*SO*_ or the pBAD33 vector were used to inoculate fresh LB medium to an initial OD_600nm_ = 0.05. Cells were grown until an OD_600nm_ = 0.2 prior to the addition of chromate at various final concentrations (0, 0.2, 0.4, 0.8 and 1.2 mM), then the cells were incubated at 30°C. The cultures were regularly sampled to measure the OD_600nm_ and to quantify the chromate using the DPC method.

*In vitro* chromate reductase activities were measured on crude extracts from *E*. *coli* cells containing either the pBAD33 vector or the plasmid p*chrA*_*SO*_ as described above for the measurements on *S*. *oneidensis* crude extracts.As ChrA_SO_ is most probably inserted inside the cytoplasmic membrane, its overexpression is toxic for the host. Preliminary growth assays of the *E*. *coli* strain harboring p*chrA*_*SO*_ were carried out at different temperatures (30°C or 37°C) in the presence of different concentrations of arabinose (0, 0.005 or 0.01%). We observed that a temperature of 30°C and the absence of inducer allowed a better growth of the strain, thus these parameters were used in further experiments.

### Construction of a deletion mutant

The *chrA*_*SO*_-deleted strain was constructed as described previously [28]. Briefly, upstream and downstream regions flanking the gene were amplified by PCR from a MR1-R strain (by using primers SO_0986 D2 to D5, S1 Table) and cloned into the suicide vector pKNG101 at the restriction sites *Bam*HI and *Spe*I. The ligation product was transformed into the *E*. *coli* CC118 λpir strain. The resulting plasmid was introduced into MR1-R strain by conjugation using the *E*. *coli* helper strain 1047/pRK2013 [29]. The plasmid was integrated in the MR1-R chromosome by a first recombination event and removed by a second recombination event in the presence of 6% sucrose. Deletion of *chrA*_*SO*_ gene was confirmed by PCR.

### Reporter gene assay

A putative promoter region was searched upstream of the start codon of *chrA*_*SO*_ using the BPROM Softberry online software (http://www.softberry.com/berry.phtml?topic=bprom&group=programs&subgroup=gfindb). A DNA fragment corresponding to the 400 bp upstream from the ATG was amplified by PCR using *S*. *oneidensis* MR1-R chromosomal DNA, digested with *Xba*I and *Spe*I, and ligated upstream of the *lacZ* gene of vector pACYC184-lacZ, which was previously constructed by cloning the β-galactosidase-encoding gene *lacZ* from pGE593 into the vector pACYC184 (S1 Table) [30,31]. The ligation product was transformed into *E*. *coli* strain MC1061. The resulting plasmid called p*chrA*_*SO*_::*lacZ* was introduced into *S*. *oneidensis* MR1-R by conjugation. Plasmid construction was checked by DNA sequencing. As a control, another fusion, named p*mxd*_*450*_::*lacZ*, was similarly constructed using a DNA fragment corresponding to the 450 bp upstream from the ATG of *mxdA* [32]. *S*. *oneidensis* MR1-R strain containing p*chrA*_*SO*_::*lacZ* or p*mxd*_*450*_::*lacZ* was grown overnight on LB-agar plate. The overnight culture was diluted to an OD_600nm_ = 0.1 in fresh liquid LB medium containing various concentrations of chromate (0, 0.05, 0.1 and 0.2 mM) and incubated in semi-aerobiosis at 28°C for 16 hours prior to β-galactosidase activity quantification. The β-galactosidase activity was determined by a Miller assay adapted for use with plate reader [33]. Briefly, cells were transferred into a microtiter plate and OD_600nm_ was measured using a Tecan Spark 10M microplate reader. Cells were then lysed using lysozyme and PopCulture reagent (Sigma-Aldrich) prior to incubation with Z buffer (62 mM Na_2_HPO_4_, 45 mM NaH_2_PO_4_, 10 mM KCl, 1 mM MgSO_4_, 50 mM β-mercaptoethanol). *O*-nitrophenyl-ß-D-galactopyranoside (ONPG) was added to the mix before kinetic quantifications of OD_420nm_. Slope of the obtained curves were calculated and β-galactosidase activity (arbitrary units) was determined by the following equation:

$$\left(1000\times slope\right)/({OD}_{600nm}\times volume\;of\;reaction(\mu L)).$$

### Bioinformatics analysis

The amino acid sequence of ChrA_SO_ was retrieved from the MicroScope Microbial Genome Annotation and Analysis Platform (https://www.genoscope.cns.fr/agc/microscope/home/). Prediction of transmembrane helices was carried out using the TMHMM online software (http://www.cbs.dtu.dk/services/TMHMM-2.0/).

Proteins sharing homologies with ChrA_SO_ were searched in the Bacteria kingdom using the BlastP software on the NCBI server (https://blast.ncbi.nlm.nih.gov). Representative sequences of different phyla were subsequently selected, as well as the sequences of four plasmid-encoded ChrA proteins that were previously characterized. For the phylogenetic analysis, we used the “Phylogeny.fr” software in the “one-click” mode, i.e. with the default parameters optimized by the authors (http://www.phylogeny.fr/). For the tree rendering step, we used the software “FigTree” version 1.4.3 (http://tree.bio.ed.ac.uk/software/figtree/) in which we entered the result in Netwick format obtained with “Phylogeny”. The tree rooting was performed with the midpoint option.

## Results

### *S*. *oneidensis* chromosome contains a chromate efflux pump encoding gene

The *S*. *oneidensis* MR-1 genome contains only one gene (*so0986*) whose product is annotated as a putative chromate efflux pump. This gene is not located on the megaplasmid but on the chromosome. Accordingly, a Blastp search performed on *S*. *oneidensis* using well-known chromate ion transporters, such as the ChrA proteins of *P*. *aeruginosa* and *C*. *metallidurans*, only identified the SO0986 protein as a ChrA homolog. In contrast, *S*. *oneidensis* does not contain homologs of the *chrB* and *chrF* genes which sometimes co-occur with *chrA*.

**Fig 1:**
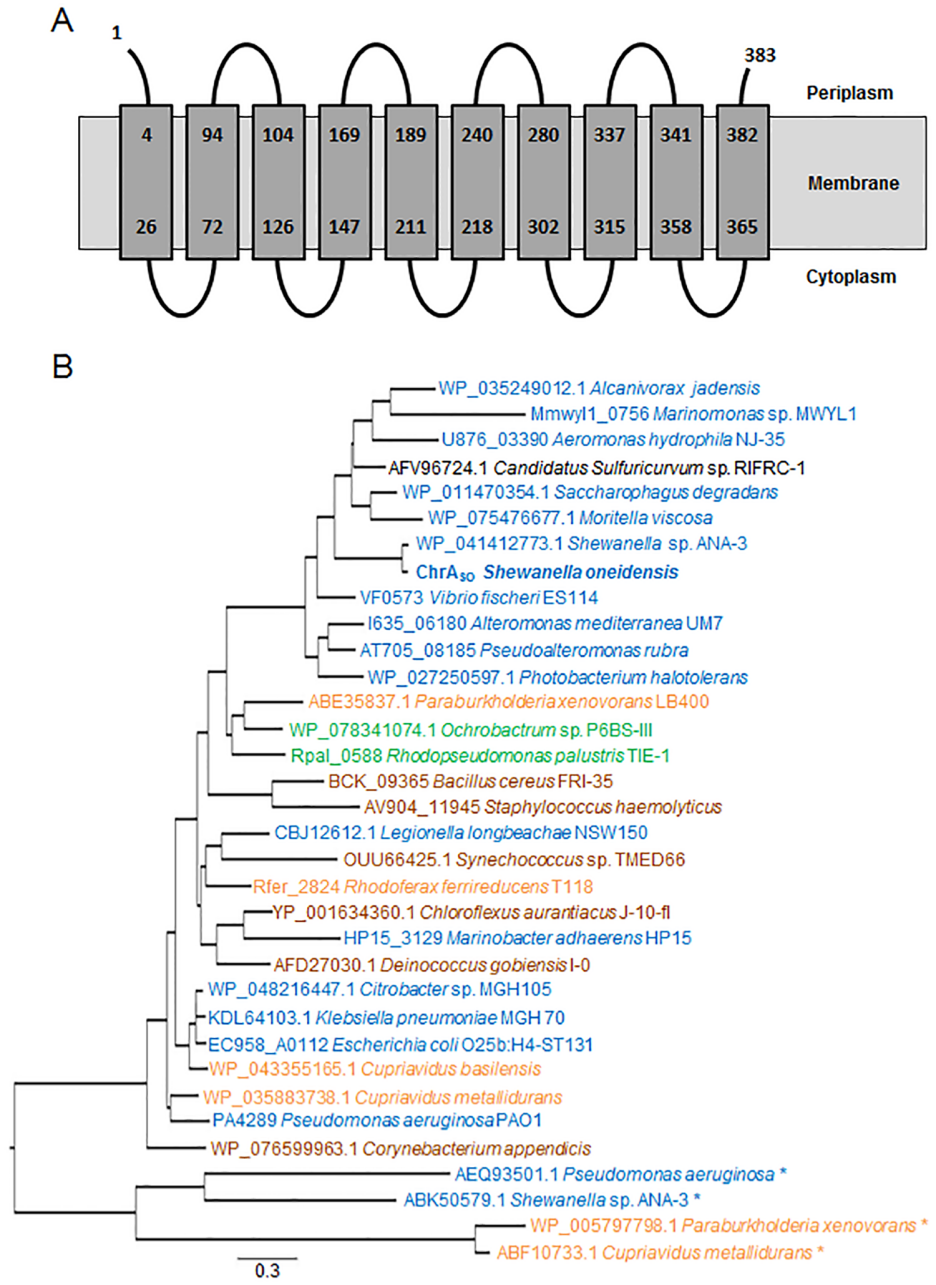
ChrA_SO_ predicted topology and phylogenetic analysis of ChrA proteins. (A) The topology of ChrA_SO_ inside the cytoplasmic membrane is schematized as predicted with the TMHMM software. The amino acids are numbered from the N- to the C-terminus of the protein. (B) Phylogenetic tree of a subset of ChrA proteins. Sequence and species names are indicated. The names are colored according to their origin: α-Proteobacteria in green, β-Proteobacteria in orange, ε-Proteobacteria in black, γ-Proteobacteria in blue and Terrabacteria in brown. ChrA_SO_ is indicated in bold. The asterisks indicate plasmid-encoded ChrA proteins.

For clarity, we will refer to the SO0986 protein as ChrA_SO_. This protein of 383 amino acids with a molecular mass of 40.9 kDa contains two chromate transporter regions (PF02417) and therefore belongs to the LCHR subfamily of the chromate ion transporter (CHR) superfamily. The TMHMM program predicted that ChrA_SO_ contains 10 transmembrane helices, which is usual for the proteins of the LCHR family. The ChrA_SO_ protein is most likely inserted in the cytoplasmic membrane with its N-terminus oriented towards the periplasm, as schematized in Fig 1A.

A Blastp search was performed using ChrA_SO_ as query. Several hundreds of homologous proteins were identified in bacteria belonging for the vast majority of them to the Proteobacteria group, but also for some of them to the Terrabacteria group. As expected, the highest percentages of identities were found between ChrA_SO_ and proteins from other *Shewanella* species. ChrA_SO_ is also highly homologous to proteins belonging in particular to *Vibrio* and *Pseudoalteromonas* species. A phylogenetic tree was constructed using a subset of proteins taken from species representative of the different taxonomic families (Fig 1B). Not surprisingly, ChrA_SO_ clusters with ChrA proteins of the γ-proteobacterial class but more particularly with those of the Aeromonadales, Alteromonadales and Vibrionales orders. Strinkigly, ChrA_SO_ shows the highest percentage of homology with the chromosome-encoded ChrA proteins from *Shewanella* sp. ANA-3, *P*. *aeruginosa* and *C*. *metallidurans* compared with their plasmid-encoded ChrA proteins, that were functionally characterized [12,13,34]. As an example, ChrA_SO_ displays 89% sequence identity with the chromosome-encoded ChrA and only 26% with the plasmid-encoded ChrA proteins of *Shewanella* ANA-3. Moreover *S*. *oneidensis* megaplasmid does not harbor *chrA* gene.

### ChrA_SO_ is involved in chromate resistance in *S*. *oneidensis*

Since ChrA_SO_ clearly belongs to the chromate ion transporter family, we wondered if it could be involved in chromate resistance in *S*. *oneidensis*. We therefore constructed a strain deleted of the *chrA*_*SO*_ gene and compared its capability to survive in the presence of chromate to that of the wild-type strain.

**Fig 2:**
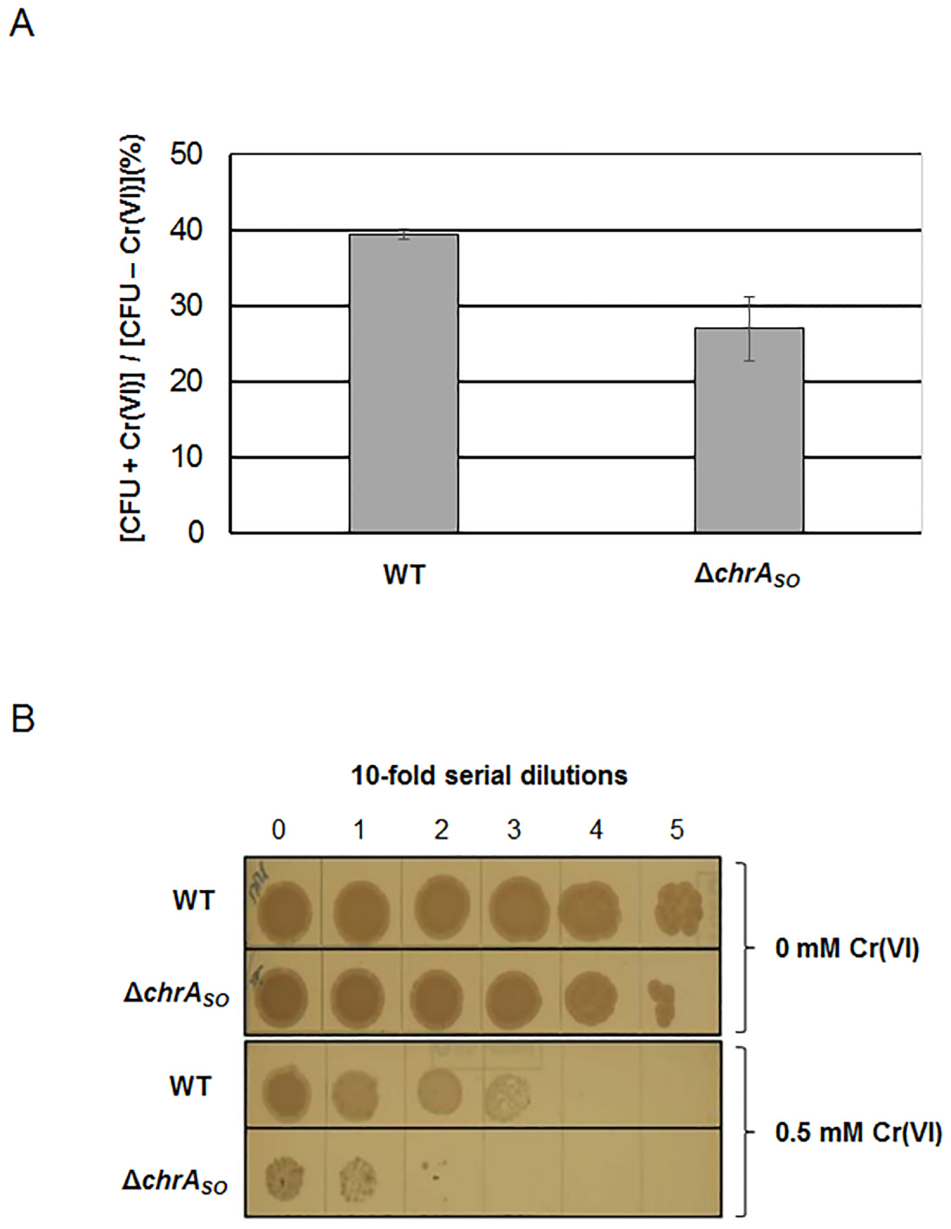
Effect of *chrA*_*SO*_ on chromate resistance in *S*. *oneidensis* strains. Two different assays were conducted to evaluate the impact of *chrA*_*SO*_ on chromate resistance. (A) In the first assay, [CFU + Cr(VI)]/[CFU—Cr(VI)] (%) indicates the percentage of viable counts of the wild-type (WT) and the *chrA*_*SO*_ deleted mutant (Δ*chrA*_*SO*_) after 5 hours of growth in semi-aerobiosis conditions and in the presence of 0.2 mM chromate, compared to that of the same strains grown in similar conditions without chromate. Values are means ± standard deviations (error bars) from at least two experiments. The mean absolute values of the number of CFU counted in the absence of chromate are 2.49 x 10^9^.mL^-1^ and 2.41 x 10^9^.mL^-1^ for the wild-type and the *chrA*_*SO*_ deleted mutant strains, respectively. (B) In the second assay, 10-fold serial dilutions of the wild-type (WT) and the *chrA*_*SO*_ deleted mutant (Δ*chrA*_*SO*_) cultures were spotted on LB agar supplemented or not with 0.5 mM chromate before incubation at 28°C during 4 days.

Wild-type and mutant cells were first allowed to grow at 28°C until an OD_600_ of 0.5. These pre-cultures were then submitted to two chromate resistance tests. In the first one, cells were allowed to grow for 5 hours after addition of 0.2 mM chromate in the pre-cultures. Afterwards, bacterial cells were diluted and plated on LB-agar to determine the percentage of survival after “colony forming unit” counting. The same experiment was carried out in the absence of chromate as a control. As observed on Fig 2A, the survival percentage is about 40% in the wild-type strain while it is only about 25% in the *chrA*_*SO*_ deleted strain. In the second test, serial 10-fold dilutions of the bacterial pre-cultures were performed and then spotted on plates containing either no chromate or 0.5 mM chromate. The plates were incubated at 28°C to allow bacterial growth. In the absence of chromate, no growth difference is observed between the wild-type and the *chrA*_*SO*_ mutant strains (Fig 2B). The presence of chromate impairs the growth of both strains, but the effect is clearly more pronounced for the *chrA*_*SO*_ mutant strain (Fig 2B). These results strongly suggest that ChrA_SO_ is involved in chromate resistance in *S*. *oneidensis*.

### Chromate reduction is impaired in a *chrA*_*SO*_ deleted strain

**Fig 3:**
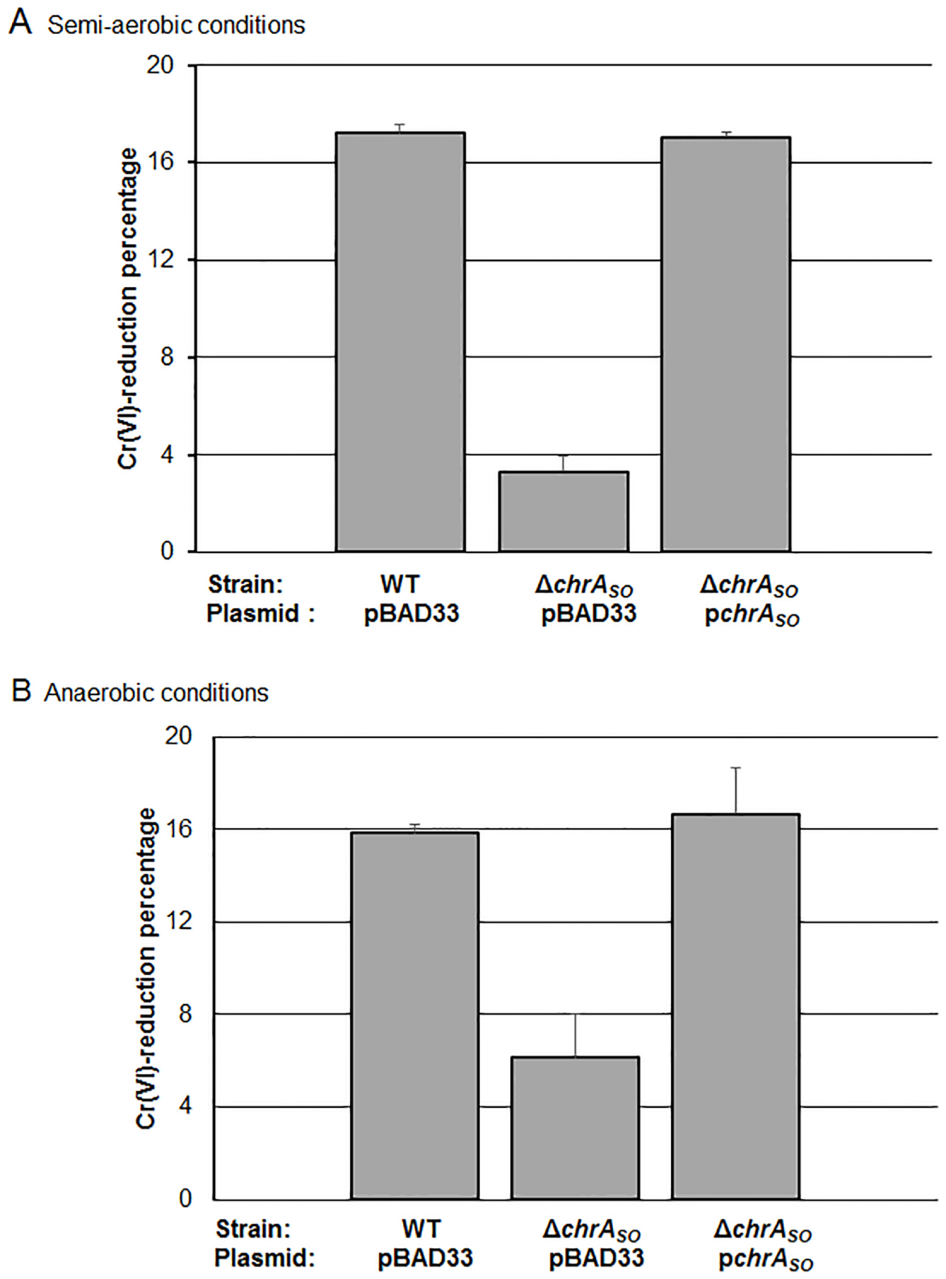
Impact of *chrA*_SO_ on chromate reduction in *S*. *oneidensis* strains. The WT/pBAD33, Δ*chrA*_SO_/pBAD33 and Δ*chrA*_*SO*_/p*chrA*_*SO*_ strains were challenged with 1 mM chromate before quantification of Cr(VI). Results were expressed as the percentage of chromate reduced after 2 hours of incubation in semi-aerobic (A) or anaerobic conditions (B). Values are means ± standard deviations (error bars) from at least three experiments.

The impact of ChrA_SO_ on the capability of *S*. *oneidensis* to reduce Cr(VI) was evaluated by comparing the percentage of Cr(VI)-reduction obtained when the wild-type, the *chrA*_*SO*_ mutant and the *chrA*_*SO*_ mutant containing the plasmid p*chrA*_*SO*_ were exposed to 1 mM chromate either in semi-aerobic (as defined in the Materials and methods section) or anaerobic conditions during 2 hours. The concentration of Cr(VI) was evaluated by the DPC method. As shown in Fig 3, in both conditions (semi-aerobiosis and anaerobiosis), the deletion of *chrA*_*SO*_ has a dramatic impact on Cr(VI)-reduction. Indeed, the amount of Cr(VI) reduced by the *chrA*_*SO*_ mutant is about one third of that reduced by the wild-type strain. As expected, the presence of p*chrA*_*SO*_ in the *chrA*_*SO*_ mutant restores a reduction efficiency comparable to that of the wild-type strain.

To decipher whether ChrA_SO_ possesses a chromate reductase activity, we performed *in vitro* chromate reductase activity on crude extracts from the wild-type and the *chrA*_*SO*_ mutant strains and the results show that the specific activity of chromate reductase is similar for both crude extracts (0.78 ± 0.1 and 0.88 ± 0.05 nmol reduced-Cr(VI).min^-1^.mg^-1^, respectively), indicating that ChrA_SO_ is not directly involved in Cr(VI)-reduction.

### The *chrA*_*SO*_ expression is induced by chromate

**Fig 4:**
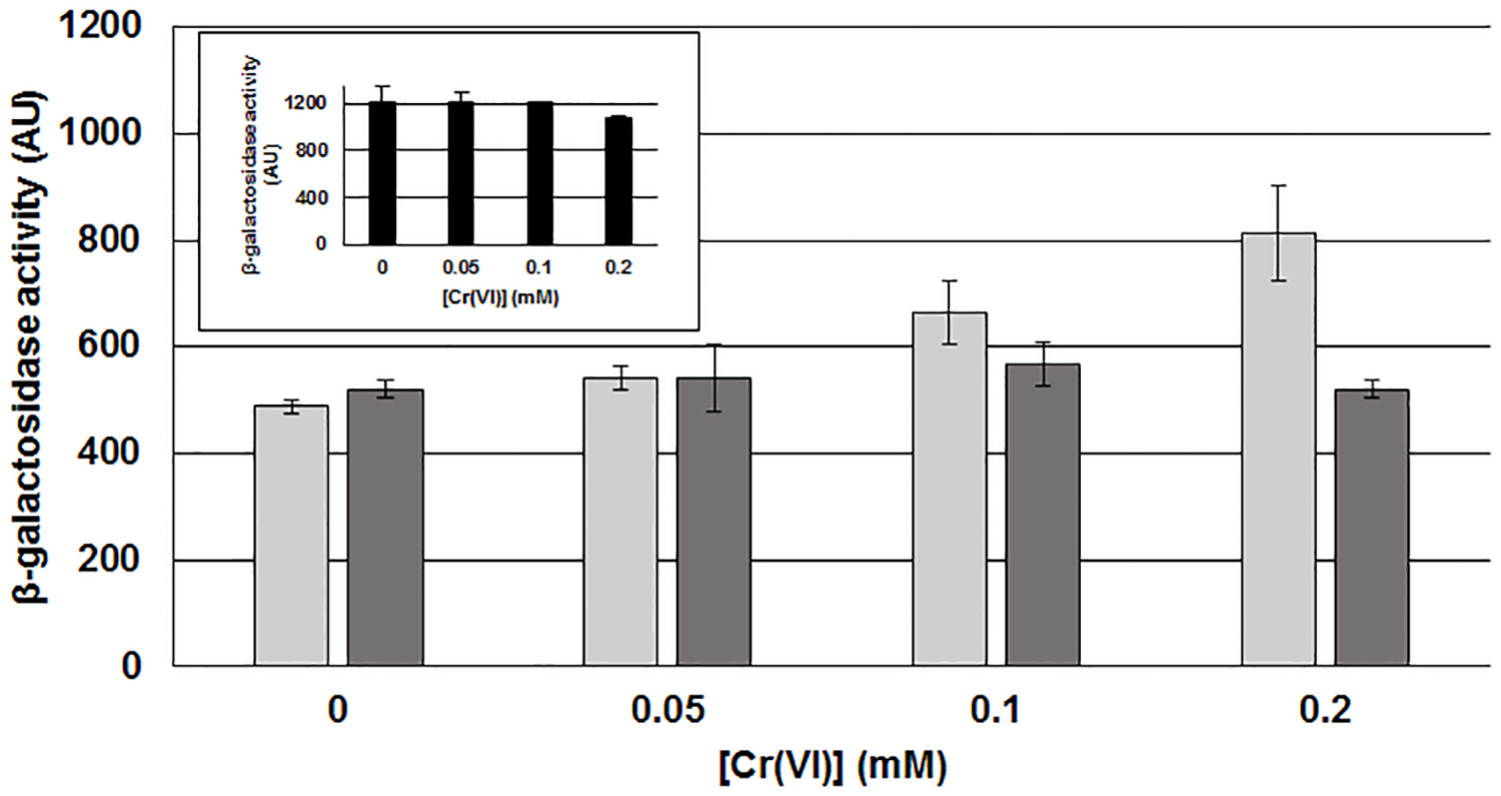
Expression of *lacZ* fusions in the presence of chromate. The wild-type *S*. *oneidensis* strain containing either the plasmid *pchrA*_*SO*_::*lacZ* (transcriptional fusion between the promoter of *chrA*_*SO*_ and the *lacZ* reporter gene; light grey bars) or the plasmid p*mxd*_*450*_::*lacZ* (transcriptional fusion between the promoter of *mxdA* and the *lacZ* reporter gene; dark grey bars), used as a control, was grown during 16 hours in the presence of increasing concentrations of chromate (0, 0.05, 0.1 and 0.2 mM) before β-galactosidase activity was determined. The MC1061 *E*. *coli* strain containing the plasmid *pchrA*_*SO*_::*lacZ* was grown in similar conditions and β-galactosidase activity was also determined (black bars in the insert). [Cr(VI)] indicates the concentration of chromate during growth. β-galactosidase activity is expressed as Miller arbitrary units (AU). Values are means ± standard deviations (error bars) from at least three experiments.

Since *chrA*_*SO*_ is involved in chromate resistance, we wondered if its expression could be regulated by chromate. We thus constructed a transcriptional fusion using the *lacZ* gene as a reporter. The sequence upstream of the start codon of *chrA*_*SO*_ was first analyzed with Bprom software (Softberry) to search for a putative promoter. Sequences corresponding to putative -10 (CATAAT) and -35 (TTTTCA) boxes separated by 20-bp were identified respectively 33 and 59-bp upstream from the start codon. The 400-bp upstream from the start codon and containing these boxes were amplified and cloned upstream of the promoterless *lacZ* gene. The plasmid bearing this transcriptional fusion was introduced in *S*. *oneidensis*. β-galactosidase activities were then measured on the resulting strain grown with various concentrations of chromate. As shown in Fig 4, a significant level of β-galactosidase is observed when the strain is grown in the absence of chromate, meaning that the putative promoter of *chrA*_*SO*_ is functional in this condition. Interestingly, β-galactosidase activity increases with rising concentrations of chromate. Indeed expression from the fusion is nearly 2-fold higher in the presence of 0.2 mM chromate than in its absence. As controls, β-galactosidase activities were measured on *E*. *coli* containing the *chrA*_*SO*_::*lacZ* fusion and on *S*. *oneidensis* containing another transcriptional fusion (*mxd*A::*lacZ*). In both cases, the activities were similar whatever the growth conditions. These results indicate that *chrA*_*SO*_ expression is induced by chromate in *S*. *oneidensis*.

### Heterologous expression of *chrA*_*SO*_ gene confers higher chromate resistance and reduction to *E*. *coli*

**Fig 5:**
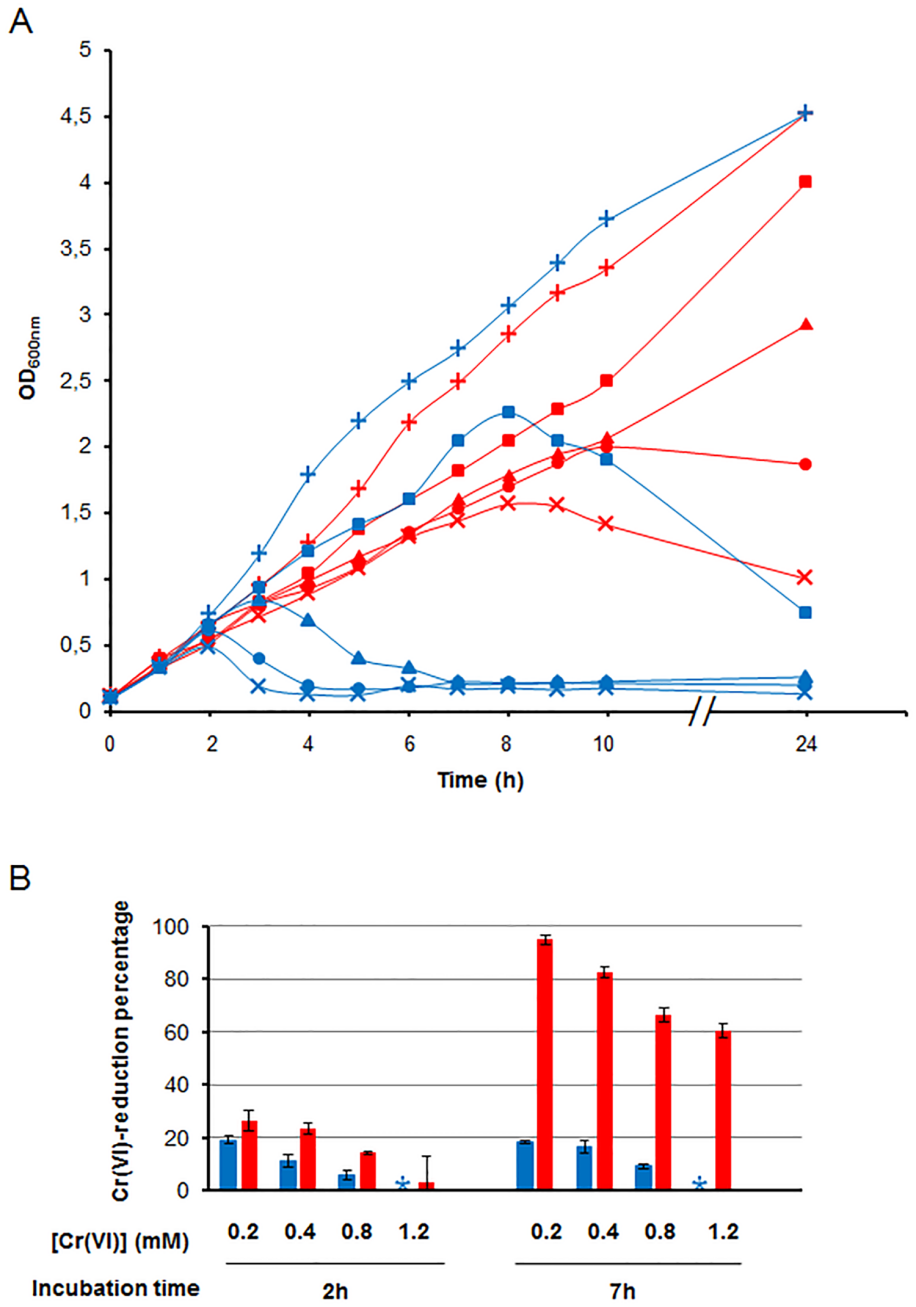
Chromate resistance and reduction by ChrA_SO_ in *E*. *coli*. (A) Chromate resistance due to the expression of *chrA*_*SO*_ in *E*. *coli* was assessed by comparing the growth of MC1061/p*chrA*_*SO*_ (red lines) to that of MC1061/pBAD33 (blue lines) in the presence of various concentrations of chromate (+, 0 mM; ■, 0.2 mM; ▲, 0.4 mM; ●, 0.8 mM and ×, 1.2 mM) at 30°C. Values are means from at least three experiments. (B) Chromate reduction by MC1061/p*chrA*_*SO*_ (red bars) and MC1061/pBAD33 (blue bars) was evaluated as the percentage of chromate reduced after 2 hours and 7 hours of challenge. [Cr(VI)], concentration of chromate added before growth expressed as mM; * indicates that the amount of chromate reduced is below detection level. Values are means ± standard deviations (error bars) from at least three experiments.

We demonstrated that *chrA*_*SO*_ plays a key role in chromate resistance and reduction in *S*. *oneidensis* and we wondered if it could play a similar role when heterologously expressed. For this purpose, *E*. *coli* strains containing either the plasmid p*chrA*_*SO*_ or the pBAD33 vector were grown in the presence of various concentrations of chromate (0, 0.2, 0.4, 0.8 and 1.2 mM) and the time course of growth was followed by measuring the OD_600_ of the different cultures. To avoid toxicity due to ChrA_SO_ overproduction, cultures were performed at 30°C without the inducer arabinose (for details see the Materials and methods section). As shown in Fig 5A, when the initial concentration of chromate is 0.2 mM, growth of both strains is similar during the first 8 hours. Then, cells containing the p*chrA*_*SO*_ continue to grow whereas cells containing only the vector stop growing. With higher initial concentrations of chromate, the growth remains possible when the p*chrA*_*SO*_ is present, although it slows down as concentration in chromate increases. In contrast, higher concentrations of chromate have a dramatic impact on cells harboring only the vector. The residual concentration of Cr(VI) was evaluated after 2 and 7 hours of growth. As shown on Fig 5B, the percentage of Cr(VI)-reduction is quickly improved when the cells contain the p*chrA*_*SO*_ compared to the ones with the vector. Reduction seems to be inefficient after 7 hours of incubation when the vector is present, whereas it is clearly improved in cells harboring the p*chrA*_*SO*_. These results suggest that ChrA_SO_ has also a positive effect on the reduction efficiency of Cr(VI) in *E*. *coli*.

We performed *in vitro* chromate reductase activity assays on crude extracts from *E*. *coli* strains containing either the pBAD33 vector or the p*chrA*_*SO*_ plasmid and the results show that the specific activity of chromate reductase is similar for both crude extracts (1.1 ± 0.12 and 1.05 ± 0.06 nmol reduced-Cr(VI).min^-1^.mg^-1^, respectively), confirming that ChrA_SO_ is not directly involved in Cr(VI)-reduction.

## Discussion

A bioinformatics analysis revealed that ChrA_SO_ (SO0986) belongs to the large family of chromate ion transporters. In agreement with this prediction, a *S*. *oneidensis* strain deleted of the *chrA*_*SO*_ gene is less resistant to chromate than the wild-type strain. Strikingly, expression of *chrA*_*SO*_ in *E*. *coli* makes the strain capable of growing in the presence of high chromate concentrations, as also observed for the plasmid-encoded ChrA of *Shewanella* ANA-3 and for several ChrA of *Burkholderia xenovorans* LB400 [34,35]. Therefore ChrA_SO_ most probably functions as a chromate efflux pump and expulses chromate ions from the cytoplasm, as demonstrated for some members of the CHR family [13,34].

It is noteworthy that, in the presence of ChrA_SO_, the percentage of Cr(VI) reduction is higher than in its absence in both *S*. *oneidensis* and *E*. *coli*. A direct role of ChrA_SO_ in Cr(VI) reduction was ruled out, since the specific activity of chromate reductase measured *in vitro* on crude extracts from both *S*. *oneidensis* and *E*. *coli* strains is similar in the absence and the presence of ChrA_SO_. One hypothesis to explain this phenomenon is that ChrA_SO_ promotes Cr(VI) reduction by expulsing chromate outside the cells where it can then be reduced. In *S*. *oneidensis*, this scenario is worth considering as two outer membrane decaheme cytochromes, MtrC and OmcA, are known to be involved in extracellular chromate reduction [21]. Another hypothesis could be that the presence of ChrA_SO_ improves cell survival by lowering intracellular Cr(VI) concentration and consequently Cr-induced damages inside the cells, which allows the cells to grow and reduce Cr(VI) over an extended period of time. This could be true for both *S*. *oneidensis* and *E*. *coli*.

This study also shows that, although *chrA*_*SO*_ is expressed without chromate, the level of expression is higher in its presence. Two previous studies performed on *S*. *oneidensis* using transcriptomic approaches missed this regulation, probably because the applied cut-offs were quite stringent [19,36]. Indeed, these studies considered only the genes showing at least a 2-fold or a 3-fold change in expression, respectively. Moreover the experimental conditions used in both studies were different from ours, which can also explain this discrepancy. The fact that *chrA*_*SO*_ expression is chromate-induced is reminiscent of what was observed for the expression of several *chr* genes in other bacteria. For example, in *C*. *metallidurans*, transcription of the plasmidic *chrA1* gene was shown to be up-regulated by chromate [12]. In *B*. *xenovorans* LB400, which possesses six genes encoding chromate ion transporters, only the *chrA2* gene present on a megaplasmid is subject to chromate-induced expression [35]. Interestingly, it was recently demonstrated that ChrA2 is the major determinant of chromate resistance in this organism [37]. The *chrBACF* operon of *Ochrobactrum tritici* 5bvl1, which is located in a chromosomally integrated transposon, proved also to be chromate-induced [38]. It was subsequently shown that the ChrB protein is a transcriptional regulator that binds to the *chrBACF* promoter region to regulate its expression [39]. As mentioned in the result section, there is no ChrB homolog in *S*. *oneidensis*, meaning that the regulation of *chrA*_*SO*_ expression probably depends on a regulatory protein belonging to a family different from that of ChrB. It will be interesting to identify this protein, since no chromate-sensing regulator except from ChrB was previously described.

In conclusion, although ChrA_SO_ plays a key role in chromate resistance in *S*. *oneidensis*, other transporters could be involved in this process. Of interest, genes encoding the three components of a putative heavy metal efflux pump (SO0518 to SO0520, CzcCBA) were observed to be up-regulated by chromate [19]. This suggests a role in chromate resistance, although this pump does not belong to the CHR family.

## Supporting information

S1 Table Plasmids and primers used in this study. (TIF)
