# Supplementary figures and images for: ChrASO, the chromate efflux pump of Shewanella oneidensis, improves chromate survival and reduction
Source: PLoS One. 2017 Nov 22;12(11):e0188516. doi: 10.1371/journal.pone.0188516 (PMC5699817; doi:10.1371/journal.pone.0188516)

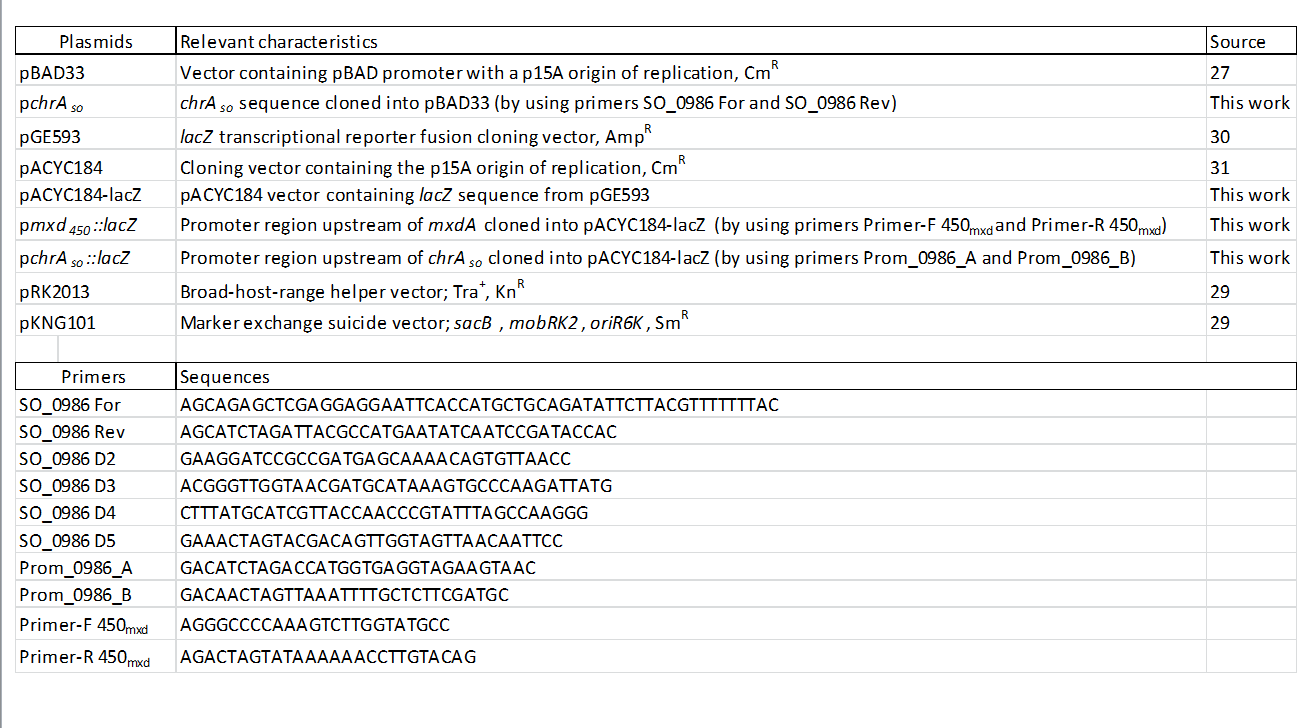

Supplement: S1 Table — (TIF) [file pone.0188516.s001.tif]
